# Smart Home Safety Technology in Medicaid LTSS: A Policy Review of HCBS Waiver Coverage

**DOI:** 10.1093/geroni/igaf122.3395

**Published:** 2025-12-31

**Authors:** Meredith Hughes, Yong Kyung Choi, Jon Pearlman, Pamela Toto, Everette James, Steven Handler

**Affiliations:** University of Pittsburgh, Pittsburgh, Pennsylvania, United States; University of Pittsburgh, Pittsburgh, Pennsylvania, United States; University of Pittsburgh, Pittsburgh, Pennsylvania, United States; University of Pittsburgh, Pittsburgh, Pennsylvania, United States; University of Pittsburgh, Pittsburgh, Pennsylvania, United States; University of Pittsburgh, Pittsburgh, Pennsylvania, United States

## Abstract

Medicaid is the largest payer for long term-services and supports (LTSS) in the United States. Though Medicaid services are subject to federal minimum standards, as well as federal review, states have some flexibility to offer new service categories in Medicaid LTSS and to define the scope of existing service categories, such as assistive technology and home environmental modifications. Smart home safety technology, is a subset of smart home technology specifically designed to enhance home-based safety by detecting and preventing home-based injuries such as falls, improving security, and supporting the aging in place of older adults. Specific examples of smart home safety technology covered in Medicaid waivers included: smart devices to control light switches, smart bulbs, electronic systems that allow individuals to control appliances and security systems, and voice activated door locks. Several states are taking advantage of this flexibility to leverage smart home safety technology to support individuals with disabilities in the home. To better understand the landscape of potential smart home technology coverage within Medicaid, a review of Section 1915(c) home and community-based services waivers covering individuals with age-related and physical disability was conducted. Section 1915(c) waivers allow for the provision of services that help Medicaid LTSS beneficiaries live independently at home, such as personal care, specialized medical equipment, and transportation. This poster will present the results of a Section 1915(c) waiver services policy scan, which looked for language indicating explicit or potential coverage of smart home safety technology within Medicaid HCBS.

